# Case report: The use of PRP in the treatment of diabetic foot: case series and a review of the literature

**DOI:** 10.3389/fendo.2023.1286907

**Published:** 2023-12-18

**Authors:** Paolo Izzo, Claudia De Intinis, Marcello Molle, Andrea Polistena, Simone Sibio, Massimo Codacci-Pisanelli, Daniele Biacchi, Pierfrancesco Di Cello, Daniele Santini, Luciano Izzo, Sara Izzo

**Affiliations:** ^1^ Department of Surgery “Pietro Valdoni”, Policlinico “Umberto I”, Sapienza University of Rome, Rome, RM, Italy; ^2^ Multidisciplinary Department of Medical-Surgical and Dental Specialties, Plastic Surgery Unit, Università degli Studi della Campania “Luigi Vanvitelli”, Naples, Italy; ^3^ Department of General Surgery, Unità Operativa Complessa (UOC) General Surgery Frosinone-Alatri at ASL Frosinone, Frosinone, Italy; ^4^ Department of Pathology, Oncology and Radiology, Sapienza University of Rome, Rome, Italy

**Keywords:** PRP, diabetic foot, wound care, ulcer, case series, literature review

## Abstract

**Background:**

Diabetes mellitus is a prevalent chronic condition that significantly impacts global health. Diabetic foot complications, such as foot ulcers, pose a substantial burden on individuals with diabetes and can lead to serious consequences, including amputation. Platelet-rich plasma (PRP) has emerged as a promising therapeutic approach for enhancing the healing of diabetic foot ulcers.

**Methods:**

In our study, we treated 12 patients with chronic diabetic ulcers using PRP injections administered at three-week intervals. Our objective was to assess the reduction in wound size and the rate of complete healing at 6 months after the start of the treatment. Additionally, we conducted a comprehensive literature review to contextualize our findings.

**Results:**

Out of the 12 patients, 8 achieved complete healing of their diabetic foot ulcers, while the remaining four showed significant improvement with more than 50% reduction in the initial lesion size. 3 patients developed mild irritation at the inoculation site. These outcomes, combined with the evidence from published studies, highlight the effectiveness of PRP in promoting the healing of diabetic foot ulcers.

**Conclusion:**

In conclusion, our study demonstrates the potential of platelet-rich plasma (PRP) as a successful therapeutic option for enhancing the healing process of chronic diabetic foot ulcers. The favorable outcomes observed, including a high rate of complete healing and significant wound size reduction, underscore the value of PRP treatment in managing this challenging complication. Further research and larger studies may provide additional insights into the mechanisms and long-term benefits of PRP in diabetic wound healing.

## Introduction

Diabetes mellitus is a prevalent chronic condition that poses significant health challenges worldwide (1). Its complications can affect multiple organ systems (causing retinopathy, renal failure and other conditions (2)), with diabetic foot complications being among the most consequential and debilitating. During the lifetime of a diabetic patient, there is approximately a 19% to 34% risk of developing a foot ulcer, with an increasing incidence in recent years due to the growing life expectancy (3). The comorbidities associated with diabetic ulcers are significant, including peripheral neuropathy, peripheral arterial disease, foot ulcers, and infections, which can lead to amputation in up to 20% of cases (4). In recent years, the use of Platelet-Rich Plasma (PRP) has increasingly gained prominence in the treatment of skin lesions and defects, finding applications not only in alopecia and skin rejuvenation but also in surgical wounds (5), scar treatment (6), and ulcers (7). PRP has become an effective tool in the treatment of these conditions, occupying a significant role in the field of dermatology, plastic surgery and wound healing. In this study, we present a series of cases involving the treatment of diabetic foot lesions using platelet-rich plasma (PRP).

## Materials and methods

We selected 12 patients between 2022 and 2023 (after obtaining informed consent) with diabetic foot ulcers that had not shown signs of healing for at least three months with conventional treatments. In addition to standard wound care (disinfection, debridement, and moist dressings), we administered perilesional platelet-rich plasma (PRP) injections during the initial outpatient visit and at a three-week interval. Patients with unstable hemodynamics due to ischemic heart disease or coagulation disorders, as well as those on anticoagulant and/or non-steroidal anti-inflammatory drug (NSAID) therapy, were excluded. Patients with the following characteristics were also excluded: platelet count below 150,000/mm³, uncontrolled diabetes mellitus (glycated hemoglobin level ≥ 75 mmol/l based on the latest laboratory data obtained within 28 days of enrollment), requirement of ongoing oral corticosteroid therapy (> 20 mg/day of prednisolone or equivalent), a history of malignant tumors with disease-free interval of three years or less and patients who are pregnant or planning to become pregnant, in order to optimize the outcome of the treatment (8). We did not make patient selections based on gender, and we did not recruit patients under the age of 18.

The product used in the study was obtained using the classical Tube Method (9). Once the blood sample (approximately 30 ml) is collected, it is gently mixed to ensure proper mixing with the anticoagulant (sodium citrate 3.8%) present in the tubes. Subsequently, two cycles of centrifugation are performed. In the first phase, the product is centrifuged at 1500 rpm for 10 minutes, resulting in the separation of the cellular components from the plasma. Using a 10 ml L/L syringe with a 22G needle, all the plasma is aspirated from the six tubes and transferred to the remaining three tubes, which are then subjected to further centrifugation at 5000 rpm for 10 minutes. The final yield of the process is approximately 3 mL of product with a volume ratio of 10:1 and a platelet concentration around ¾ times higher than whole blood (ranging from 1.1 × 10^6^ to 1.7 × 10^6^ platelets/μl).

The treatment of the wound is divided into several phases. Before proceeding with wound disinfection using iodopovidone, it is crucial to assess the presence of bacteria in the wound through a qualitative swab. The presence of an infection represents a negative prognostic factor and can compromise the response to treatment. In case the culture examination is positive, targeted antibiotic therapy will be administered based on the antibiogram. In the second phase, after thorough disinfection, surgical debridement (Wound Bed Preparation) is performed, involving the removal of non-vital tissues until a clean and bleeding wound bed is achieved. Subsequently, a second disinfection is carried out. The actual treatment involves the infiltration of 3 ml of PRP onto the wound bed and along the wound’s edge, using a 1 ml syringe and a 30G needle. At the end of the procedure, the ulcer is covered with a non-adherent dressing, using non-adherent gauzes.

This procedure is repeated once three week when changing the dressing until the healing of the wound or for a maximum of 12 weeks.

The dimensions (based on the greatest diameter) of the lesions were evaluated upon entry into the study and at a three-month interval after the last injection. Additionally, the rate of complete wound healing (defined as the absence of any breaks in the epidermis) was quantified (10). The ulcers were assessed using the PUSH scale (11) at the beginning of the treatment (T0)and at the 6-month follow-up(T6).

We then conducted a literature search on PubMed and Scopus using the following search terms: “PRP AND diabetic foot”, “PRP AND diabetic ulcer”, “Platelet rich plasma AND diabetic foot”, “Platelet rich plasma AND diabetic ulcer”.

## Results

Out of the 12 treated patients, 4 were male and 12 were female. The age ranged from 65 to 81 years, with a mean of 76 years and a median of 78 years. The average size (based on the greatest diameter of the lesion) at the beginning of the treatment was 2.17 cm (with a median of 2 cm and a range between 1 cm and 4.4 cm). None of the ulcers treated was gangrenous according to the Wagner Score (see **Table 1**) (12). Six patients had high blood pressure, and five met the criteria for metabolic syndrome (defined as having three or more of the following five criteria: waist circumference over 40 inches (men) or 35 inches (women), blood pressure over 130/85 mmHg, fasting triglyceride (TG) level over 150 mg/dl, fasting high-density lipoprotein (HDL) cholesterol level less than 40 mg/dl (men) or 50 mg/dl (women), and fasting blood sugar over 100 mg/dl) (13).

**Table 1:** Cumulative data of the patients treated.

	Patients treated (n=12)
Sex	
- Male	4
- Female	8
Age (years)	
- Mean	76
- Median	78
- Range	65-85
BMI	
- Mean	-26.4
- Median	-27
- Range	-23-28.5
HbA1c (mmol/l)	
- Mean	67 mmol/l
- Median	65 mmol/l
- Range	57-73 mmol/l
ABI (Ankle/Brachial Index)	
- Mean	0.94
- Median	0.8
- Range	0.6-1.2
Comorbidities	6 patients with clinical hypertension
	5 with metabolic syndrome
Wagner Score	- 8 (grade 2)
	- 4 (grade 3)
Distribution of the ulcers	- 2 hindfoot
	- 6 midfoot
	- 4 forefoot
Push score T0	
-Mean	6
-Median	6
-Range	3-10
Push Score T6	
-Mean	1.23
-Median	0
-Range	0-4
Dimension of the lesion before the treatment (Cm)	
-Mean	2.17
-Median	2
-Range	1-4.4
Dimension of the lesion after the treatment (Cm) (counting complete healing as 0 cm)	
-Mean	0,4
-Median	0
-Range	0-1,2
Rate of complete healing	66,67% (8/12)
Adverse effects	3 mild irritations at the inoculation site

**Figure 1:**
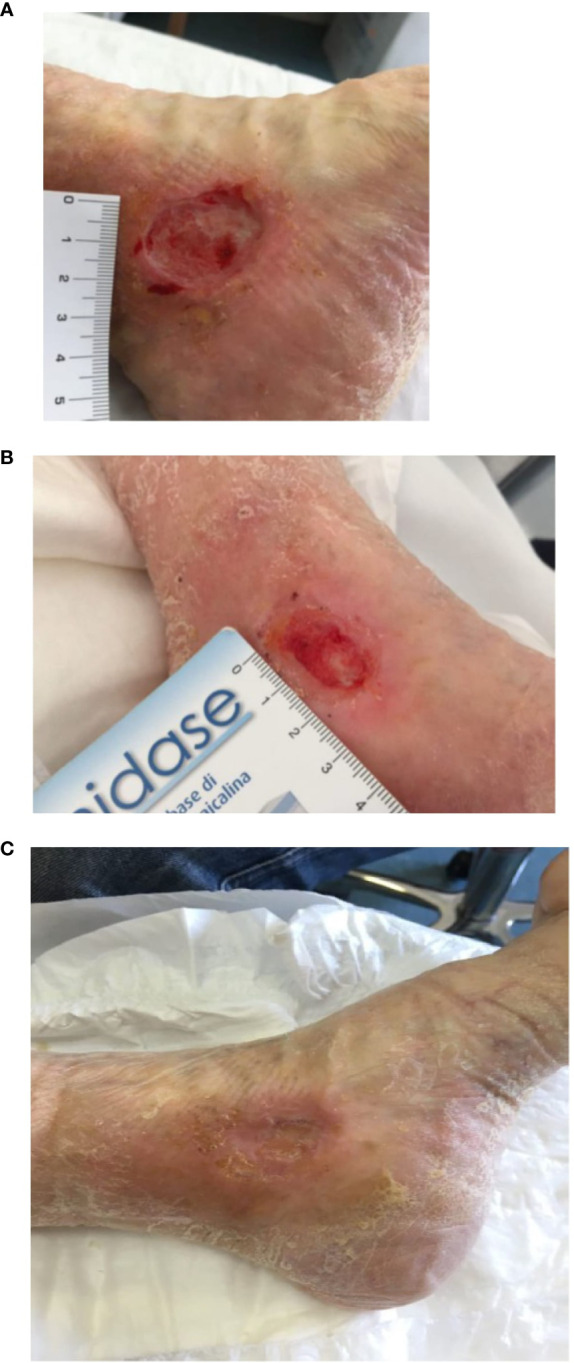
Patient with peri malleolar ulcer before the treatment **(A)**, after the first PRP injection **(B)** and at the end of the treatment **(C)**.

At the end of the treatment, 8 patients achieved complete healing with restitution ad integrum of the skin (with a complete healing rate of 66.67%), while the remaining four subjects experienced partial healing (>50% reduction in size of the lesion), with an average size of 1.1 cm (median 1.1 cm, range 1-1.2 cm) (**Table 1**) (**Figures 1A–C**). No major adverse effect or infections of the lesions were observed during the treatment, 3 patients developed mild irritation at the inoculation site.

## Discussion

The use of PRP has been introduced in clinical practice for approximately 20 years (14–19), although the first studies analyzing this technique date back to the 1990s (20–23).

From the early studies, the efficacy of using plasma derivatives has demonstrated significant effectiveness in accelerating recovery time and wound healing speed. Patients treated with plasma derivatives showed a weekly healing rate of 0.4 cm2 compared to 0.13 cm2 in non-treated patients (22). In 2009, a systematic review conducted in Brazil (24) analyzed 18 studies, including 7 randomized clinical trials. The review highlighted a significant effect on the healing process (95% CI odds ratio 2.94-20.31). However, it also noted a lack of consistency in the type of treatment and protocol used, as well as variability in the type of dressings and study design (25). This variability among the studies has made it difficult to establish a unified standard in this field of research and reflects the considerable challenges in implementing standardized protocols for the treatment of difficult wounds. During the same period, a prospective randomized trial conducted in Greece (26) compared the healing of large difficult wounds (>2.5 cm in any one dimension) using a protease-modulating matrix with or without the addition of platelet-derived growth factors. The study did not find statistically significant differences in accelerating the healing process between the two groups. Another interesting application of PRP was tested in an Italian study in 2009 (27), where PRP was used as an adjuvant to autologous adipose tissue grafting in the treatment of lower limb ulcers and cervical facial defects. This combined treatment demonstrated efficacy both *in vitro*, where it increased the survival of adipose cells, and *in vivo*, with complete healing of all lower limb lesions achieved in an average of 9.7 weeks. A study conducted in 2015 (28) evaluated the use of PRP in the treatment of diabetic foot ulcers in patients with lower limb vascular diseases. The study included 72 patients, 30 of whom had Critical Limb Ischemia (CLI). The patients were treated for a period of 24 months, and the results showed a reduction in ulcer area of >90% in 52 patients and a limb salvage rate of 89% (100% in patients without CLI, 73% in patients with CLI). These findings demonstrated the effectiveness of PRP even in challenging patients with multiple comorbidities. Results similar to those have been obtained from a Japanese study (29). During the same period, other studies have demonstrated an improvement in the healing process through the use of PRP in diabetic foot ulcers (30–35). Other studies, on the other hand, have considered the antibacterial effect of PRP (30) to reduce the incidence of superinfections in the lesions (36, 37). Recently, new methods have been devised to deliver the growth factors in PRP, such as in a 2020 Chinese study where an injectable hydrogel with Platelet-Rich Plasma release was developed (38). Other studies have evaluated the efficacy of PRP treatment based on the administration vehicle, and they have found that the most effective mode of administration remains perilesional injection. Indeed, in a study from 2023, patients treated with topical PRP gel showed slower healing rates compared to those treated with perilesional injections (39).

The action of PRP in wound healing appears to be caused by the presence of numerous growth factors (such as TGF-B, PDGF, FGF, and ECGF), chemical mediators like histamine and serotonin, and proteins such as CTAP-3 and PF4, with effects on angiogenesis and the activation of extracellular matrix (ECM) cells. The existence of these growth factors, particularly when VEGF and FGF-2 are concurrently present (40), triggers the development of new blood vessels at the injection site, as observed in both animal models (41) and *in vivo* studies (42). Additionally, the presence of VEGF seems to enhance blood flow (42). Additionally, PRP has antibacterial properties. These combined mechanisms are the basis for PRP’s action in wound healing (43–45).

## Conclusion

Platelet-rich plasma (PRP) has been widely used in the treatment of diabetic foot and associated wounds, demonstrating effectiveness and utility over time, along with a reduced rate of complications. Recent biotechnological advancements are opening new avenues in its delivery and the development of combined therapies, thus leading to increasingly exciting prospects in regenerative medicine.

## Data availability statement

The datasets for this article are not publicly available due to concerns regarding participant/patient anonymity. Requests to access the datasets should be directed to the corresponding author.

## Ethics statement

Written informed consent was obtained from the participant/patient(s) for the publication of this case report.

## Author contributions

PI: Writing – original draft, Writing – review & editing. CD: Writing – original draft, Writing – review & editing. MM: Writing – original draft, Writing – review & editing. AP: Data curation, Writing – original draft. SS: Data curation, Writing – original draft. MC: Data curation, Writing – review & editing. DB: Methodology, Writing – review & editing. PD: Data curation, Writing – original draft. DS: Conceptualization, Writing – review & editing. LI: Methodology, Writing – review & editing. SI: Writing – original draft, Writing – review & editing.
